# Advances in Multi-Scale Mechanical Characterization of Materials with Optical Methods

**DOI:** 10.3390/ma14237282

**Published:** 2021-11-28

**Authors:** Luciano Lamberti

**Affiliations:** Dipartimento di Meccanica, Matematica e Management, Politecnico di Bari, 70125 Bari, Italy; luciano.lamberti@poliba.it

The mechanical characterization of materials embraces many different aspects, such as, for example, (i) to assess materials’ constitutive behavior under static and dynamic conditions; (ii) to analyze material microstructure; (iii) to assess the level of damage developed in the material; (iv) to determine surface/interfacial properties; and (v) to optimize manufacturing processes in terms of process speed and reliability and obtain the highest quality of manufactured products.

Multi-scale analysis is the most correct approach to the mechanical characterization of materials because “macroscopic” behavior represents the average of the statistically distributed behaviors exhibited by material elements at the micro- and nanoscales. Most of the investigation techniques currently available are suited for a specific scale and rarely allow the achievement of a comprehensive overview of material behavior. Optical methods (OMs) are naturally suited for the multi-scale mechanical characterization of materials [1,2] in view of their capability to accurately measure displacements, strains and stresses in real time and to gather full-field information without altering specimen conditions. OMs cover a fullrange of wavelengths from X-ray to visible light and infrared. Non-conventional illumination and super-resolution techniques may increase measurement resolutions to the nanoscale. A definite strength of OMs is the possibility of changing measurement scales by properly modulating wave frequencies and choosing parameters of experimental setups. For example, the sensitivity of moiré techniques can pass from small to large deformations by simply changing the pitch of the grating used in the measurements.

The basic principle of OMs is very simple [1,2]: light wavefronts hit the specimen surface and are then modulated by the deformed body’s surface. By comparing the wavefronts recorded by a sensor before and after the body’s deformation, a system of fringes is obtained; each fringe is the locus of an iso-displacement region. The spatial frequency of fringes is directly connected to the strain field. The displacement information may be extracted from a deterministic signal or from a random signal. Moiré techniques follow the modulation of a regularly spaced grating applied or projected onto the specimen surface. Speckle techniques deal with a random signal modulated by the variation of surface roughness’ distribution caused by the deformation. Image correlation techniques follow the evolution of a random pattern printed on the specimen surface caused by loading.

This Special Issue focuses on the multi-scale mechanical characterization of materials with optical methods. This Special Issue includes nine research articles covering the assessment of constitutive behavior and determination of stiffness properties of materials, the identification of failure stages/mechanisms, the measurement of residual stresses, microstructure and/or surface analysis, failure analysis of industrial components and quality control of manufacturing processes.

Ficarella et al. [3] presented a hybrid framework for the mechanical identification of materials and structures. Displacement fields measured with intrinsic/projection moiré and speckle interferometry setups were compared with their counterparts computed by finite element models reproducing the experiments. The difference between measured and computed displacements was then minimized by means of three advanced metaheuristic optimization algorithms. This strategy was selected because displacement fields represent the direct solution of the general elasticity problem. The algorithms were enhanced to reduce the computational cost of the identification process. Three test cases including up to 17 unknown parameters were successfully solved in [3]: (i) the determination of elastic constants of a composite laminate used as a substrate in electronic boards; (ii) the mechanical characterization and layup identification of an axially compressed composite panel for aeronautical use; and (iii) the determination of hyperelastic constants and reinforcement fiber orientation of bovine pericardium patches used in the biomedical industry.

Sciammarella et al. [4] experimentally verified the concept of the representative volume element (RVE). A given volume of material of size *L* may include different microcomponents of size *d*; strain values are statistically distributed with respect to the *L/d* ratio but converge to a deterministic value for the RVE. The study of [4] originated from the fact that the outcome of OM experimental observations may strongly depend on the selected RVE’s scale. The concept of RVE was hence framed in a very comprehensive context connecting image analysis, kinematics of deformation, the material’s structure, constitutive behavior and failure mechanisms. The connection between different scales was illustrated by the analysis of an Al–SiC composite material with a metal matrix reinforced by hard particles. The second example presented in [4] regarded the identification of the constitutive model of urethane rubber. Moiré techniques were used for measuring displacement fields in both examples.

Yoshida and Sasaki [5] monitored the deformation status of a tensile specimen made of aluminum alloy. Using the symmetry principle of physics, Yoshida [6] defined in his previous studies the criteria for the different stages of deformation (linear elastic, plastic and fracturing stages), expressed by certain spatio-temporal features of the differential displacement generated during a small time interval. In [5], differential displacement maps were obtained from Electronic Speckle Pattern Interferometry (ESPI), and the interferometric fringe patterns were interpreted based on the criterion for each deformation stage. Remarkably, the criteria of linear elastic deformation, plastic deformation and fracturing stage clearly appeared in the corresponding ESPI fringe patterns, consistently with the loading characteristics.

The determination of residual stress with optical methods (i.e., ESPI and digital image correlation) is discussed in Refs. [7,8]. In particular, Sasaki et al. [7] evaluated changes in elastic modulus caused by residual stresses. Compressive or tensile residual stress should, respectively, increase or decrease elastic modulus. In the calibration phase, a steel plate specimen was gripped in a tensile loading machine and temperature was locally increased to further expand the material. However, the machine’s grips limited the specimen’s expansion and hence the apparent thermal expansion per unit increment of temperature was lower than for free thermal expansion (i.e., unconstrained specimen). Thermal deformation was measured by an ESPI optical setup sensitive to 2D in-plane displacements for different tensile loads. Hence, the apparent thermal expansion coefficient was correlated to the initial stress level. The same local heating and ESPI measurement protocols were used for dissimilar joints of steel and cemented carbide plates prepared by butt-brazing. The evaluation of residual stresses was proven feasible using the visualization of reversible thermal deformation in the temperature range of ±10 °C.

Chen et al. [8] used digital image correlation (DIC) to measure residual stress in Ag and ZrN thin films. DIC served to analyze the SEM images obtained through incremental ring-core drilling performed with a focused ion beam (FIB) at various depth steps. The milling operation deforms the specimen and the full-field displacements of each SEM image analyzed by DIC correspond to the difference between the whole image and the reference SEM image recorded before milling. The FIB-DIC procedure was accurately calibrated to accurately measure residual stresses. Residual stresses of Ag and ZrN thin films measured with FIB-DIC were compared with X-ray diffraction (XRD) results. While FIB-DIC and XRD measurements agreed quite well, the former approach has fewer restrictions on external conditions and material selection. Furthermore, FIB-DIC may be used for full-field analysis and has a wider application range than other methods.

Optical methods can provide useful information on surface topography, surface damage, crack sizes and distribution, as well as interface properties between different materials. In this regard, Shi et al. [9] analyzed the relationship between surface topography and friction properties of a rough contact interface (6016 aluminum alloy/CoCrMo) under fluid dynamic pressure lubrication conditions. Surface topography parameters indicated in the ISO 25178 international standard (i.e., surface height arithmetic mean *S*a, surface height distribution kurtosis *S*ku, surface volume average volume *V*vv and surface center area average void volume *V*vc) were measured using an optical microscope profilometer and a scanning electron microscope. Friction tests were then carried out with a multifunctional tribometer and the effects of surface topographic parameters on friction were analyzed, and the wear mechanism of the worn surface was assessed.

Li et al. [10] analyzed the relationships between wear resistance, microstructure and processing parameters for high-carbon 8 mass% Cr tool steel. In order to improve the wear resistance of tools, steel matrix composites are synthesized by spraying carbide on the metal surface. The distribution of carbides strongly affects wear resistance, but the effect of tempering temperature on carbide precipitation and wear resistance for high-carbon 8 mass% Cr tool steel is not completely understood. In [10], optical microscopy was utilized to analyze the microstructure of specimens tempered at different temperatures. SEM microscopy then detected individual carbides and carbides nucleated on oxides. Electron-probe microanalysis finally showed the segregation of alloying elements. Friction tests were then carried out and data on mass loss were related to tempering temperature and microstructure composition.

Optical inspection may contribute to improving industrial production processes. For example, Jamil et al. [11] investigated the main causes for the rejection of sugar mill roller shafts after rough machining. Non-destructive (optical microscopy, SEM microscopy, X-ray spectroscopy, ultrasonic and magnetic particle testing) and destructive micro- and macro-crystallographic tests were carried out for this purpose. In particular, optical microscopy inspection of surfaces revealed that the as-cast structure of the shaft contains ferrite within a fine pearlite matrix and at prior austenite grain boundaries, as well as alumina and oxide-type inclusions. SEM inspection further verified the inclusions. The microstructure was related to the formation of macro-surface cracks and subsurface microcracks observed via non-destructive testing. A root cause analysis (RCA) approach highlighted the refractory lining, the hot-top of the furnace and the ladle as significant causes of the presence of inclusions in the shaft microstructure. Consequently, these parts were replaced with a better quality refractory material. The feedback statistics, evaluated over more than one year, indicated that the rejection rate of shafts could be reduced by up to 7.6%.

Singh et al. [12] investigated the machinability and optimized turning parameters (i.e., cutting speed, feed rate, depth of cut) of the difficult-to-cut pure Ti alloy. A new cooling system, namely vortex tube-assisted minimum quantity lubrication (VMQL), was introduced and then evaluated by performing 26 experiments with different selected combinations of turning parameters. The surface roughness of machined workpieces, tool flank wear, cutting forces and power consumption were measured for each experiment. Optical microscopy was used to inspect the worn tool surface. Experimental data were fitted by response surfaces, and metaheuristic optimization algorithms and desirability methods were utilized to find the best combination of turning parameters.

The variety of topics covered by this Special Issue demonstrates without a shadow of a doubt the high versatility of optical methods that may be applied practically in all fields of mechanical engineering. OMs have reached a very high level of standardization, regardless of the investigation scale. This makes OMs the best choice for optimizing all stages of a product’s life, starting from material characterization, passing through design verification, and ending with monitoring/improvement of the production process and the evaluation of product quality.
